# PhyloM: A Computer Program for Phylogenetic Inference from Measurement or Binary Data, with Bootstrapping

**DOI:** 10.3390/life12050719

**Published:** 2022-05-11

**Authors:** Sudhindra R. Gadagkar

**Affiliations:** College of Graduate Studies (Biomedical Sciences Program), College of Veterinary Medicine, Midwestern University, Glendale, AZ 85308, USA; sgadag@midwestern.edu; Tel.: +1-623-572-3855

**Keywords:** measurement data, binary data, pairwise distances, neighbor-joining, phylogenetic inference, algorithm, computer program, bootstrapping, rooting, open source

## Abstract

Quantitative and binary results are ubiquitous in biology. Inasmuch as an underlying genetic basis for the observed variation in these observations can be assumed, it is pertinent to infer the evolutionary relationships among the entities being measured. I present a computer program, PhyloM, that takes measurement data or binary data as input, using which, it directly generates a pairwise distance matrix that can then be subjected to the popular neighbor-joining (NJ) algorithm to produce a phylogenetic tree. PhyloM also has the option of nonparametric bootstrapping for testing the level of support for the inferred phylogeny. Finally, PhyloM also allows the user to root the tree on any desired branch. PhyloM was tested on Biolog Gen III growth data from isolates within the genus *Chromobacterium* and the closely related *Aquitalea* sp. This allowed a comparison with the genotypic tree inferred from whole-genome sequences for the same set of isolates. From this comparison, it was possible to infer parallel evolution. PhyloM is a stand-alone and easy-to-use computer program with a user-friendly graphical user interface that computes pairwise distances from measurement or binary data, which can then be used to infer phylogeny using NJ using a utility in the same program. Alternatively, the distance matrix can be downloaded for use in another program for phylogenetic inference or other purposes. It does not require any software to be installed or computer code written and is open source. The executable and computer code are available on GitHub.

## 1. Introduction

Quantitative measurements and binary data are routinely collected in biological experiments and field investigations. The data often take the form of a table or matrix with the subjects of the study (species or other taxonomic entities) and the measurement or binary criteria (morphometric measurements, results of metabolic tests, etc.) in corresponding rows and columns. This information can then be used for direct comparisons, for example, to help establish the uniqueness of one or more taxa, perhaps as an argument for the discovery of one or more new species (e.g., [1]).

**Figure 1:**
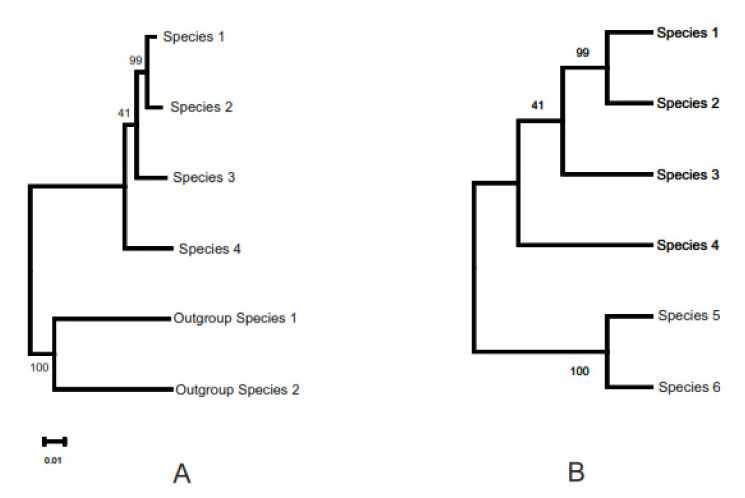
Depiction of a phylogenetic tree in two different ways. (**A**) Rooted tree with branch lengths, and (**B**) topology only. The differences and implications of these depictions are explained in the text.

Phylogenetic inference has also become routine in biology and is used in numerous contexts, such as tracing the evolutionary history of a group of species or determining the relationships among them, studying migration routes, understanding and predicting outbreaks of viral infection, etc. The result of phylogenetic inference takes the form of a line graph made up of branches and nodes (Figure 1). Each node represents a divergence or speciation event, and thus, the beginning of two (if bifurcating) or more new lineages. In Figure 1A, the length of a given branch (horizontal line) reflects the amount of evolutionary change in that lineage that has occurred between two consecutive nodes as one traverses the tree from left to right. Such a depiction of the tree is always accompanied by a scale that helps calibrate the length of any branch in the tree in terms of units of evolution. On the other hand, each branch length merely serves to connect two nodes in Figure 1B and does not represent a biologically relevant event or phenomenon. The latter type of tree thus only depicts the topology or the relationships among the taxa, with no information about the amount of evolution that has taken place between two nodes connected by a branch. It is, therefore, not accompanied by a scale.

The tree in Figure 1A is also “rooted.” In other words, it shows the history of divergence or speciation events. It must be noted that the tree in either depiction in this figure (A or B) can be rooted. The rooting of a tree refers to anchoring the tree using one or more taxa that are known to belong to a taxonomic group outside (typically evolutionarily closely related but basal to) the one that the species of interest belong to (hence, “outgroup”). For example, if the species of interest are placental mammals, an ideal outgroup would consist of one or more taxa of marsupials and perhaps monotremes. The presence of a root in the tree is indicated by the small, open-ended branch on the left, which connects the tree to other, even more basal taxa that have not been included in the analysis. The tree in Figure 1B has no direction, and the root of the tree can be placed on any branch, making that node ancestral to the tree.

As a final point of explanation, the number by each node in both depictions reflects the percentage of support in the data for that particular divergence event, obtained by a process known as nonparametric bootstrapping [2,3], where the data used in inferring the tree are resampled many times (100 or more) with replacements, and the phylogeny is inferred using the resampled data each time with the same method as for the original tree, finally noting the number (percentage) of times each node in the original tree is seen among the bootstrap replicate trees.

With the availability of rapid and cost-effective DNA sequencing technologies, the reconstruction of phylogenies based on molecular data has become ubiquitous in biology, and there are a number of sophisticated computer programs available, such as MEGA [4]; PHYLIP [5]; PAUP [6]; MrBayes [7], etc., used by expert users and non-specialists alike. There are also numerous methods by which phylogenies can be inferred, such as neighbor-joining (NJ), Maximum Likelihood (ML), Maximum Parsimony (MP), and Bayesian Inference (BI), each with its own optimality criterion.

Molecular phylogenetic inference begins with obtaining nucleotide or amino acid sequences for one or more markers of interest (e.g., genes) for each of the taxa in the dataset and aligning them such that they are rendered suitable for correct site-by-site comparison across the sequences. The phylogeny can be inferred using any of the above methods, all of which use either discrete characters (nucleotides or amino acids in the alignment) or pairwise evolutionary distances among the sequences in the alignment as input [8]. In character-based methods, each character is considered among all the sequences in the alignment simultaneously, while in distance-based methods, evolutionary distances are calculated for each pair among all the taxa in the group of interest and arranged in the form of a pairwise distance matrix. In either case, the evolutionary relationships are inferred based on an optimality criterion specific to the method.

Least squares (LS) and Minimum Evolution (ME) are two distance methods of phylogenetic inference. The neighbor-joining (NJ) method, developed by Saitou and Nei in 1987 [9], is an efficient and extremely popular algorithm of the ME method. The NJ method is a cluster algorithm that begins with a star tree (where all the terminal nodes are equidistant from each other). The process of inferring the phylogeny proceeds by first selecting a pair of taxa to join based on the least distance between them in the pairwise distance matrix. The pairwise distances among all the remaining taxa are then recalculated, accounting for the two taxa already joined. NJ thus continues to join pairs of taxa or nodes in a hierarchical manner, until a fully resolved phylogeny is obtained. While simulation-based studies have shown that ML outperforms NJ when there is a large proportion of missing data [10,11], simulations have also shown that NJ is very efficient in inferring large trees [12], whether the evolutionary rates are equal or unequal [13], and does equally well in inferring shallow or deep branches [14].

Pairwise distances among the species can be obtained from a molecular sequence alignment by counting the number of sites with different nucleotides/amino acids in each pair of sequences.

This information is better captured in the form of the number of changed sites as a proportion of the sequence length. This is known as the *p* distance [15]. While the *p* distance is a valid measure of evolutionary distance between two taxa, its utility lies mainly in recently diverged taxa and diminishes for taxa that have greater times of divergence. This is because the alignment tends to become “saturated,” that is, it reaches a state when every nucleotide site in every sequence has mutated once. After saturation, no further mutations are captured by the *p* distance. In order to account for these “invisible” mutational changes, one needs to model the rate of nucleotide substitution. Many such models have been developed and are routinely used when inferring phylogenies for taxa that are not recently diverged [16].

While molecular phylogenetic inference encapsulates the evolutionary relationships among taxa based on sequence changes in ever more sophisticated ways [8], species evolve in ways that are not necessarily reflected in the sequences, and therefore, these changes cannot be captured by molecular phylogenetics. One salient example is functional changes, especially those resulting from regulatory innovations. The significance of regulatory control over gene function was recognized by Susumu Ohno [17], even though he had championed gene duplication as the means of introducing functional novelty. Subsequently, King and Wilson also concluded that the phenotypic differences between two species are unlikely to be captured by a comparison of molecular sequences [18]. Perhaps then, rather than relying on molecular sequence data to model the evolution of functional changes among the group of taxa being investigated, methods to capture the variation in gene expression levels are necessary. There are various methods that do so, such as microarrays, RT/PCR (qRT/PCR), Northern Blotting, and RNA-Seq. However, as shown by Ding et al. [19], the results appear to be a function of the methodology used. In any event, knowledge of the genetic basis of expression variation is necessary in order to know which genetic markers to target by these methods. Emerson and Li [20] suggest that the variation in regulatory control among species can be directly estimated by means of integrating QTL mapping, the *cis/trans* component of gene expression variation, and population genetics methodologies. There have been since been multiple studies carried out to help understand the patterns of regulatory control of gene expression divergence (e.g., [21,22,23]). However, to my knowledge, there has been no attempt at elucidating the evolutionary relationships among species using regulatory control or gene expression level as a trait to build a phylogeny on. A practical solution to addressing this question is to use objective and measurable outcomes as data, such as the results of a metabolic panel among various bacterial taxa.

Here, I present a computer program, PhyloM (Phylogenetic Inference for Measurement data), that first calculates the distance matrix directly from the data using a simple algorithm and then uses this distance matrix to infer the phylogeny. In addition to basic phylogenetic inference, PhyloM also has the option of non-parametric bootstrapping to provide statistical support for the tree. A further option is the ability to root the tree on a user-defined branch (outgroup). Apart from measurement data, PhyloM can also use raw binary data (0/1 or +/−) as the input matrix (for which the algorithm to calculate the pairwise distance matrix is different; see below).

PhyloM is an open-source Windows program (with the source code available on GitHub (https://github.com/sgadagkar/PhyloM (accessed on 22 March 2022)) for download, modification, cross-compiling to other platforms, etc.). It has a graphical user interface (GUI) that can be run entirely by pointing and clicking. Furthermore, since the program is made available as an executable binary, there is no need to install anything. PhyloM has been written with the express purpose of making a user-friendly program available to the end-user who is typically without the expertise to write computer code or does not have the time or inclination to do so. It is being made available in the same spirit of being of service to biologists as in an earlier program of mine, HEPB [24], which is very popular because of its ease of use and because it asks of no expertise from the user other than pointing and clicking on a GUI.

**Figure 2:**
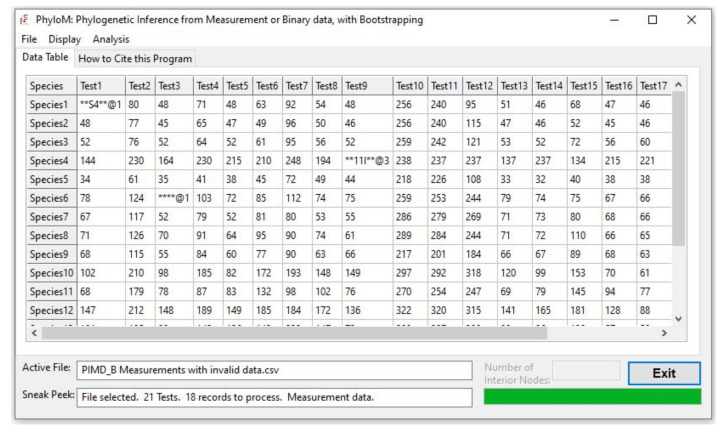
The main GUI of PhyloM showing the input data in the form of a grid. Invalid data are highlighted by means of a pair of asterisks on either side of the offending data and a pointer (@) to the specific character/digit. For example, the entry for Species1, Test1 has a letter (S) at the first character, the cell at Species4, Test9 has a letter (I) at the third character, and finally, the cell at Species6, Test3 is blank. Note the other useful information provided to the user in the interface.

PhyloM first converts the measurement or binary data into a pairwise distance matrix, where the distances are observed pairwise differences in the data among the taxa, in a manner that is equivalent to the *p* distances for molecular sequences explained in Figure 2 below. Thus, the recommendation for PhyloM is that it be ideally used to infer the phylogeny among closely related taxa.

Sophisticated computer programs do exist that can be used to infer phylogenies using measurement data (e.g., [25,26]), but they require the user to install software packages, learn new programming languages, and write computer code, the expertise for which is typically lacking among most biologists.

## 2. Materials and Methods

PhyloM is a Windows-based computer program, written using the Lazarus IDE [27], incorporating the following algorithms:

### 2.1. Algorithm for Computing the Pairwise Distance Matrix from Measurement Data

In general, for a measurement matrix of values (***X***) for *m* species (rows) and *k* tests (columns), the pairwise difference, *δ,* between Taxa *i* and *i* + 1 for Test *j* is computed, in a stepwise manner (in *m* − 1 steps), as:

(1) $$\delta_{\left(i,i+1\right),j}=|X_{ij}-X_{i+1,j}|$$

and the pairwise distance, *D* (defined as the average of the differences between the two taxa across all *k* tests), is given as:

(2) $$D_{i,i+1}=\frac{1}{k}\sum_{i}^{k}\delta_{\left(i,i+1\right)}{}_{,j}$$

The marginal pairwise distances are then compiled into a triangular matrix.

### 2.2. Algorithm for Computing the Pairwise Distance Matrix from Binary Data

Consider a matrix ***X***, of binary (+/− or 0/1) responses of *m* subjects (e.g., species) to *k* tests. An individual cell’s value, *λ*, is first obtained in the following manner:

(3) $$\lambda_{\left(i,i+1\right),j}=\left\{\begin{array}{l} 0,\;\mathrm{if}\;X_{ij}=X_{(i+1),j}\\ 1,\;\mathrm{if}\;X_{ij}\neq X_{(i+1),j} \end{array}\right.$$

Next, the pairwise distance, *D*, between taxa *i* and *i* + 1 is obtained as in Equation (2) across the *k* tests. As before, all the marginal pairwise distances are compiled into a triangular matrix.

Note that *D* (computed using either algorithm) is conceptually similar to the pairwise distance *p*, the observed proportion of sites that are different between two sequences in the context of a molecular sequence alignment and phylogenetics (see above).

### 2.3. Genome Tree Reconstruction

Phylogenetic inference was carried out using the Codon Tree method with default settings in PATRIC version 3.6.7 [28]. This method selects single-copy global Protein Families (PGFams) [29] as homology groups from the PATRIC genome sequence database and analyzes aligned proteins and coding DNA from single-copy genes using the program RAxML [30]. Protein sequences are aligned using MUSCLE [31]. A concatenated alignment of the nucleotides and proteins is written to a Phylip-formatted file, and then into a partitions file for the RAxML algorithm [30,32]. This file describes the alignment in terms of the proteins, followed by the first, second, and third codon positions. Support values were generated with 100 rounds of ‘Rapid bootstrapping [33] of RAxML. All genomic data are available in the NCBI GenBank database and can be located using the accession numbers: PPTF00000000, MKCR000000000, PQWB00000000, JZJL00000000, JZJJ00000000, NC005085, MQZZ00000000, JAJOHW000000000, JYKA00000000, and RFAR00000000.

### 2.4. Metabolic Measurement Data

Data for the phenotypic tree were generated with a Biolog Gen III system (Biolog, Hayward, CA, USA) using medium A in a Biolog microstation as described by the manufacturer. Each isolate was independently tested at least twice. Measurement data recorded at 24 h were normalized by subtraction of the negative control well and averaged. Negative data were assumed to have a value of ‘0’.

## 3. Results

PhyloM was designed and developed for the express purpose of providing a freely available, user-friendly, menu-driven computer program for visualizing the evolutionary relationships manifest in measurement or binary data matrices. PhyloM uses data input by the user to first compute a pairwise distance matrix. The program then infers the phylogeny using neighbor-joining [9], with options for rooting the tree and bootstrapping. PhyloM has a number of unique features that assist the user with easily understood and contextual feedback, as explained below.

### 3.1. Input File

The input file needs to be a comma-delimited (.csv) file with rows (records) for taxa and columns (fields) for the recording criteria. PhyloM can accommodate numerical measurements as well as binary (0/1 or +/−) recording criteria. The user clicks on File | Open in the main menu in PhyloM, which brings up a window, Select Data Type, which informs the program about the type of data being imported. Upon successfully importing the data file, the GUI opens up a tab, Data Table. This tab displays the data in the form of a grid for easy and efficient inspection. Any formatting errors in the data trigger a message and an error log that can be saved. In addition, the user can view the error(s) in the Data Table tab where the offending cells are flagged by means of a pair of asterisks flanking the data, with a pointer to the specific character (Figure 2).

### 3.2. Distance Matrix

After successfully importing an error-free data file, the user clicks on Analysis|Make Distance Matrix in the main menu to compute the pairwise distance matrix. This produces a new tab, Distance Matrix, where the pairwise distances can be viewed in the form of a lower triangular matrix in a grid. If the user wishes to use the distance matrix in another program, there are several format choices to save the file in: .csv format, MEGA format (for use in MEGA [4]), or NEXUS format (for use in programs such as PAUP [6], Mesquite [34], etc.)

### 3.3. Phylogenetic Inference

After the pairwise distance matrix has been computed, the user can click on Analysis|Make Tree in the main menu for phylogenetic analysis using the neighbor-joining method [9]. After clicking on the OK button in the subsequent Information message, another window opens up named Bootstrap Options. Checking the checkbox brings up the option of leaving the number of bootstrap replicates at the default of 100 or changing it to any desired number. Upon clicking on OK, the inferred tree is displayed in the Tree Viewer.

**Figure 3:**
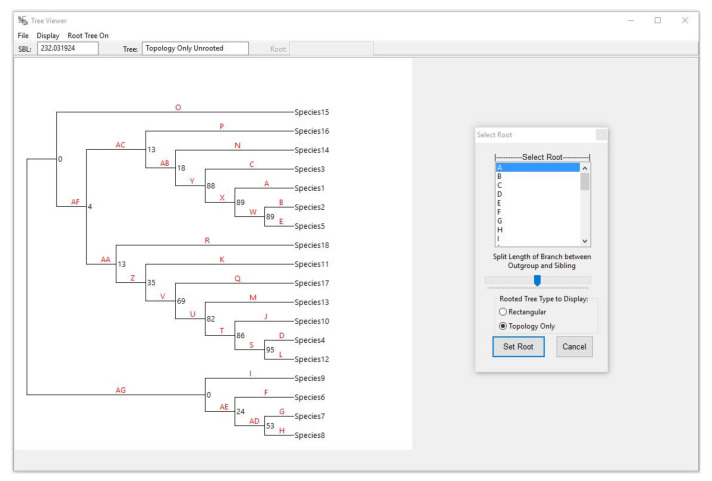
The Tree Viewer form of PhyloM showing the inferred tree with bootstrap values. Shown also is the Select Root utility that becomes available when the Root Tree On menu item is clicked on. This action also shows the topology of the tree and labels all the branches with letters in red font for easy selection of the root branch. A given branch can be chosen by clicking on the corresponding letter in the Select Root window. See text for explanation of the various utilities available in the Tree Viewer and Select Root windows.

Tree Viewer has several functionalities built into it. The File menu allows for saving the tree as an image (jpeg) or text (Newick format) file. Clicking on Root Tree On brings up the options to root the tree by changing the rectangular tree to a topology-only tree for easy viewing, assigning a label to each branch, and simultaneously bringing up another window named Select Root. This latter window lists all the branch labels and allows the user to select one to root the tree on. The tree with the labeled branches can be viewed on the side for easy branch selection (Figure 3).

**Figure 4:**
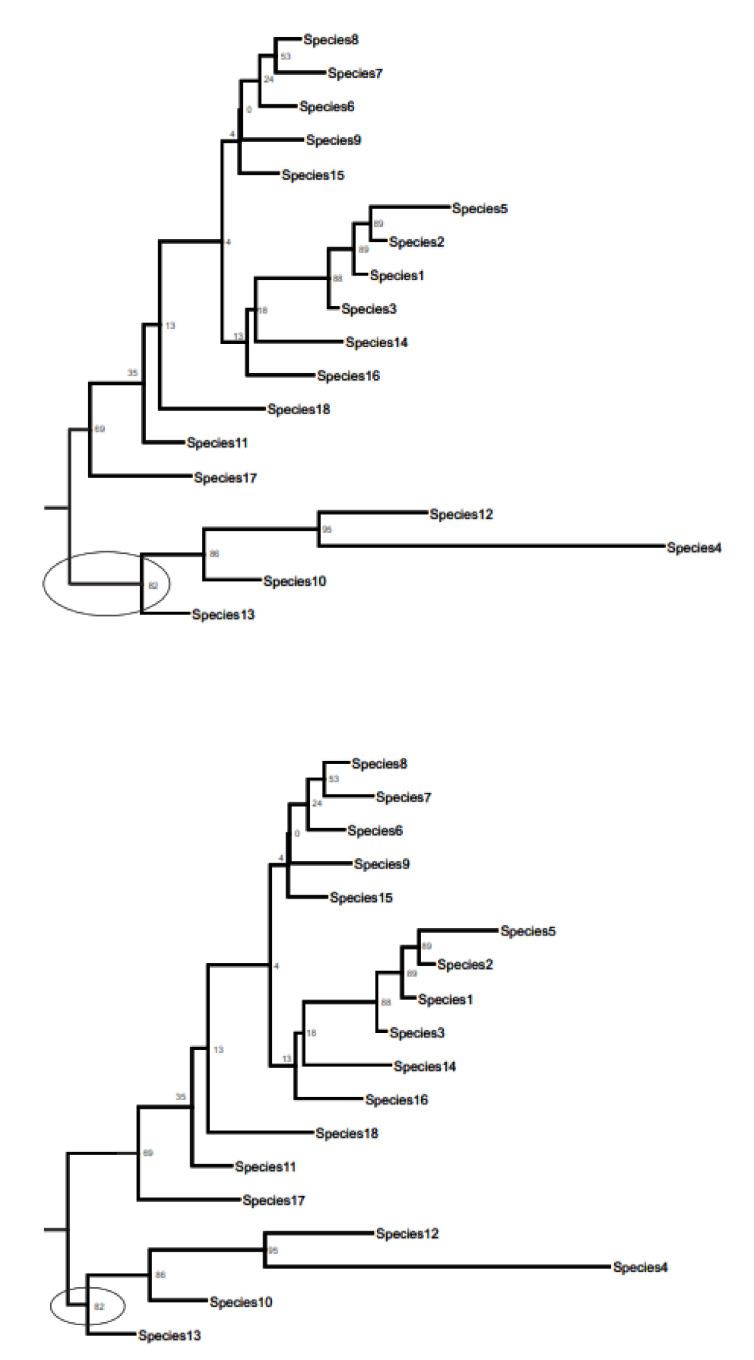
Figure showing the effect of moving the slider in the Select Root window (Figure 3), which can be used to determine where the root is placed on the branch connecting the outgroup and its sibling lineage in the tree. See text for more details.

The Select Root window also provides the option to set the root at a desired point along the length of the branch that the user chooses to root the tree on. This is illustrated in Figure 3, where the tree on the top has the slider in Split Length … Sibling almost all the way to the left, and the tree on the bottom has the slider close to the right end of the slider. Note the difference in the length of the branch for the common ancestor for each of the two affected clades between the two trees (encircled branches; Figure 4).

This feature is useful if there is information on the proportional times before the diversification of the two sister clades after splitting from the root and can be useful for computing or calibrating times of divergence. The Select Root window also contains the option to root the original, unrooted tree without changing the topology. (Note that the original tree, inferred using neighbor-joining, is unrooted.) This is done by selecting the innermost branch as the root (the last in the list of branches under Select Root). After making the desired choices, clicking on the Set Root button then roots the tree on the selected branch, which is now displayed on the left. The Display menu in Tree Viewer has four options that allow the user to display the tree or only the topology in a rooted or unrooted fashion. Tree Viewer also displays SBL (the sum of branch lengths), Tree (the type of tree currently being displayed), and Root (if rooted, the label of the branch that the tree is rooted on).

## 4. Discussion

PhyloM was conceived to fulfill a need expressed by colleagues who sought to understand the evolutionary signal in measurement or binary data that they had collected on a group of species, apart from the evolutionary signal manifest in the molecular sequences of the species in question. Thus, PhyloM was developed not to replace phylogenetic inference from molecular sequences, but rather to understand the evolutionary signal in the measurement or binary data. The evolutionary relationships evidenced by such a tree may or may not be congruent to the tree derived from molecular sequence data. Assuming that the latter reflects the true evolutionary history of the species in question, the tree obtained using the measurement/binary data should produce an interesting comparison with the species tree, possibly revealing similarities among species across large divergence times and differences between closely related species. Such an understanding of the evolutionary relationships among species with respect to the measurements is revealed by inference of the phenetic relationships among the taxa (as done by PhyloM), and an understanding of the evolution of these relationships is best understood when the two trees are compared.

**Figure 5:**
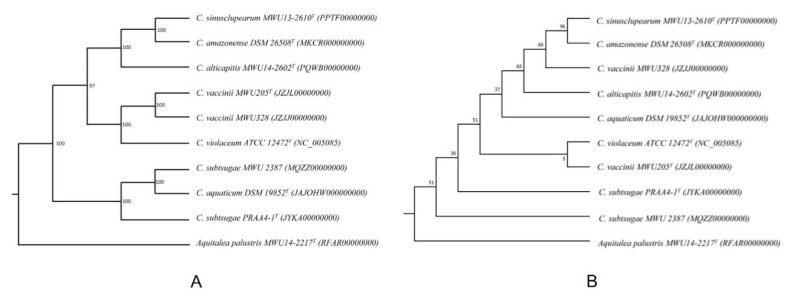
(**A**) Phylogenetic inference using whole-genome sequences of nine *Chromobacterium* species/strains and one outgroup (*Aquitalea* sp. MWU14-2217) in PATRIC. GenBank accession numbers are shown in parentheses. Details of how the tree was inferred are given in the text. (**B**) Phylogenetic inference using measurement (BIOLOG) data from nine *Chromobacterium* species/strains and one outgroup (*Aquitalea* sp. MWU14-2217) using PhyloM. GenBank accession numbers are shown in parentheses.

I illustrate the utility and importance of phylogenetic inference employing measurement data using ten bacterial (nine *Chromobacterium* species/strains and a strain of *Aquitalea* sp. as an outgroup) genome sequences and the corresponding Biolog Gen III measurement data (Table S1) generously provided by Scott Soby of Midwestern University. The genomic RAxML tree indicates the relationships among nine members of the genus and the outgroup using randomly selected single genes from the PATRIC Global Protein Families [28], and can be seen in Figure 5A, which also contains the GenBank accession numbers of the species/strains used.

The phenotypic tree for the same taxa inferred from BIOLOG data using PhyloM, which depicts the expression of a range of metabolic, stress response, and antibiotic resistance genes under identical growth conditions, shows a different set of relationships within the genus (Figure 5B).

Both trees group *C. amazonense* DSM 26508^T^ and *C.* sp. MWU13-2610 together, implying that their net behaviors with respect to the various tests in BIOLOG are congruent with their genotypic evolution. On the other hand, *C. aquaticum* CC-SEYA-1^T^, which is grouped with the two *C. subtsugae* strains (MWU 2387 and PRAA4-1^T^) in the genotypic tree (Figure 5A), is in a completely different clade when phenotypic evolution is considered (Figure 5B). The two *C. vaccinii* strains are also separated in the phenotypic tree into different clades. While *C. vaccinii* MWU205^T^ groups with *C. violaceum* ATCC 12472^T^ with very low bootstrap support in the phenotypic tree, it is rather distantly connected to *C. vaccinii* MWU328 in this tree.

**Figure 6:**
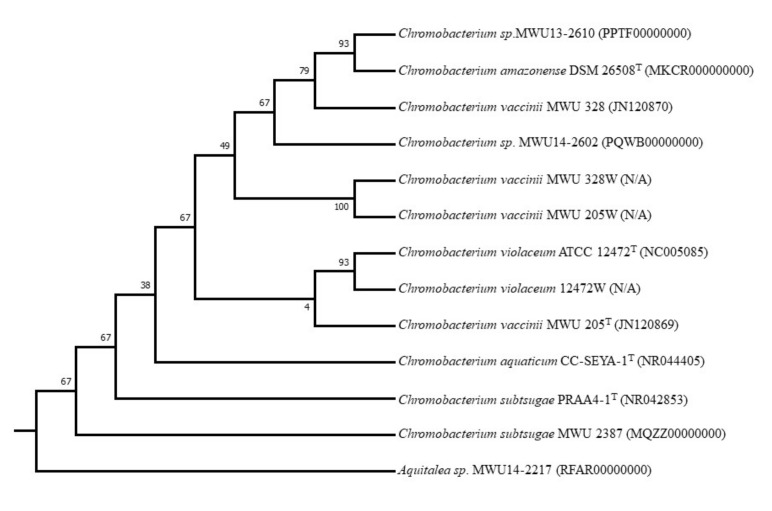
Phylogenetic inference using measurement (BIOLOG) data from nine wild-type *Chromobacterium* species/strains, three quorum-sensing mutants, and one outgroup (*Aquitalea* sp. MWU13-2217) using PhyloM. GenBank accession numbers are shown in parentheses, except for the QS mutants.

Figure 6 plots the phenotypic tree of the same ten taxa, but with the addition of quorum-sensing mutants of three taxa: *C. vaccinii* MWU 205, *C. vaccinii* 328, and *C. violaceum* 12472 (their QS mutant status designated by a “W” for “White”—lacking the violacein pigment that their wild-type versions produce [1]). Although the QS mutation is the result of only one non-synonymous mutation in the quorum-sensing receptor gene (*cviR*), the BIOLOG-based phenotypic tree does not group the wild-type and corresponding mutants together, indicating that the loss of metabolic gene expression due to the inability to sense the quorum-sensing signal is a key component of their phenotypic identity. The addition of three cognate quorum-sensing mutants indicates how the loss of a key global gene regulator can alter the expression, and therefore the relationship, of members of the genus. Interestingly, in the case of *C. violaceum* ATCC 12472, the mutant strain does not markedly differ from the wild type in its metabolic gene expression.

Instances of congruence and incongruence between the genotypic and phenotypic trees can inform the researcher about parallel evolution in specific traits, and as illustrated by the loss of quorum-sensing, regulatory elements within the organism. However, the use of a tool such as PhyloM in conjunction with molecular phylogenetics can reveal parallel evolution with respect to a whole suite of connected traits, as shown here.

PhyloM is an open-source, GUI-based, user-friendly, Windows-based computer program that is intended for use by the biological community for determining evolutionary relationships among members of a group of taxa for which measurement or binary data are available for a number of criteria. The computer code is freely available on GitHub (https://github.com/sgadagkar/PhyloM (accessed on 22 March 2022)) and can be freely downloaded to make modifications or for cross-compilation on other platforms, if desired. The distance matrix generated by PhyloM was input into MEGA [4], and the phylogeny was inferred using neighbor-joining. PhyloM and MEGA produced identical trees in comparable times. The advantage of PhyloM over MEGA or any phylogenetic software is that, in addition to inferring the phylogeny using NJ with bootstrapping and rooting, it produces a distance matrix directly from measurement or binary data in an extremely user-friendly GUI, using only pointing and clicking, with nothing to install and no computer code to learn or write.

## Data Availability

Not applicable.

## Supplementary Materials

The following supporting information can be downloaded at: https://www.mdpi.com/article/10.3390/life12050719/s1. Table S1. BIOLOG data obtained for nine *Chromobacterium* species/strains, one outgroup species, *Aquitalea* sp. MWU14-2217, and three quorum-sensing *Chromobacterium* mutants. The data have been normalized by subtracting the value of the corresponding negative control value. Details regarding BIOLOG can be found in the text. Where available, GenBank accession numbers are given in parentheses.
